# Heparan sulfate proteoglycans (HSPGs) and chondroitin sulfate proteoglycans (CSPGs) function as endocytic receptors for an internalizing anti-nucleic acid antibody

**DOI:** 10.1038/s41598-017-14793-z

**Published:** 2017-10-30

**Authors:** Hyunjoon Park, Minjae Kim, Hye-Jin Kim, Yeonjin Lee, Youngsil Seo, Chuong D. Pham, Joungmin Lee, Sung June Byun, Myung-Hee Kwon

**Affiliations:** 1 Department of Biomedical Sciences, Graduate School, Ajou University, 206 World cup-ro, Yeongtong-gu, Suwon, 443-749 South Korea; 2 Department of Microbiology, Ajou University School of Medicine, 206 World cup-ro, Yeongtong-gu, Suwon, 443-749 South Korea; 3 Animal Biotechnology Division, National Institute of Animal Science, RDA, 1500, Kongjwipatjwi-ro, Iseo-myeon, Wanju-gun, Jeollabuk-do 565-851 South Korea

## Abstract

A subset of monoclonal anti-DNA autoantibodies enters a variety of living cells. Here, we aimed to identify the endocytic receptors recognized by an internalizing anti-nucleic acid autoantibody, the 3D8 single-chain variable fragment (scFv). We found that cell surface binding and internalization of 3D8 scFv were inhibited markedly in soluble heparan sulfate (HS)/chondroitin sulfate (CS)-deficient or -removed cells and in the presence of soluble HS and CS. 3D8 scFv colocalized intracellularly with either HS proteoglycans (HSPGs) or CSPGs in HeLa cells. 3D8 scFv was co-endocytosed and co-precipitated with representative individual HSPG and CSPG molecules: syndecan-2 (a transmembrane HSPG), glypican-3 (a glycosylphosphatidylinositol (GPI)-anchored HSPG); CD44 (a transmembrane CSPG); and brevican (a GPI-anchored CSPG). Collected data indicate that 3D8 scFv binds to the negatively charged sugar chains of both HSPGs and CSPGs and is then internalized along with these molecules, irrespective of how these proteoglycans are associated with the cell membrane. This is the first study to show that anti-DNA antibodies enter cells via both HSPGs and CSPGs simultaneously. The data may aid understanding of endocytic receptors that bind anti-DNA autoantibodies. The study also provides insight into potential cell membrane targets for macromolecular delivery.

## Introduction

Proteoglycans, a large heterogeneous group of heavily glycosylated proteins, comprise a core protein and one or more covalently attached glycosaminoglycans (GAGs)^1^. Proteoglycans are classified into several distinct groups according to the nature of the GAG(s) on the core protein. In general, they possess a single type of GAG chain, such as heparan sulfate (HS), chondroitin sulfate (CS), and dermatan sulfate (DS), on serine residues of the core protein and are designated HS proteoglycans (HSPGs), CS proteoglycans (CSPGs), or DS proteoglycans, respectively^1^. In particular, HSPGs and CSPGs are thought to be receptors/co-receptors for a variety of ligands and to function in cellular signaling.

In HSPGs and CSPGs, both HS and CS are highly negatively charged GAGs due to acidic sugar residues and/or modification by sulfate groups. Their synthesis begins with the covalent attachment to specific serine residues on the core protein in the Golgi apparatus. HS chains up to more than 100 sugar units long are linearly polymerized by the addition of alternating glucuronic acid (GlcA) and N-acetyl-glucosamine (GlcNAc) residues and are extensively modified. Modifications to the GlcA-GlcNAc disaccharide unit include N-deacetylation and N-sulfation of GlcNAc, epimerization at C-5 of GlcA into iduronic acid (IdoA), which results in an HS chain composed of repeating disaccharide units of IdoA-GlcNAc, and various sulfations such as O-sulfation at C2 (2 S) of GlcA and IdoA, O-sulfation at C6 (6 S) of GlcNAc and N-sulfated glucosamine (GlcNS), and O-sulfation at C3 (3 S) of N-glucosamine (GlcN) residues. A CS chain is a linear polymer comprising repeating units of GlcA and N-acetylgalactosamine (GalNAc) disaccharides. CS chains also undergo modification, such as epimerization and sulfation, which generate structural complexity. Epimerization of GlcA to IdoA within the polymer generates DS disaccharide units along the CS chains, resulting in hybrid CS/DS chains. Depending on the number and location of sulfate groups on the disaccharide units of CS (GlcA-GalNAc) and DS (IdoA-GalNAc), their fine structures are classified into the six units: O, A, C, D, B, and E for CS chains, and iO, iA, iC, iD, iB, and iE for the corresponding DS chains. For example, CS-A, CS-C, or DS has A (GlcA-GalNAc-4S), C (GlcA-GalNAc-6S), or iA (IdoA-GalNAc-4S) unit, respectively, as the major disaccharide unit, but also contains other disaccharide units as minor components^1–6^.

HSPGs expressed on the surfaces of human cells are classified into four syndecans (SDCs), which are integral membrane proteoglycans, and six glypicans (GPCs), which are attached to the cell surface via a glycosylphosphatidylinositol (GPI) anchor^3,5^. HSPGs act as internalizing receptors and/or as co-receptors for temporary cell surface attachment to promote internalization of a variety of macromolecules such as DNA, cationic polymers, liposomes^7^, cell-penetrating peptides (CPPs)^8^, viruses^9–12^, protein aggregates^13^, RNases^14,15^, and cancer cell exosomes^16^. In the case of CSPGs, most are secreted from cells and serve as extracellular matrix molecules that are widely expressed in the developing and adult central nervous system; however, several CSPGs are expressed on cell surfaces^17^. Cell surface CSPGs can be either transmembrane (e.g., CD44, NG2 (also known as CSPG4) and RPTP-σ), or GPI-anchored (e.g., GPI-brevican (BCAN, also known as CSPG7)). In contrast to the numerous documents regarding endocytosis via the binding of macromolecules to HSPGs, the reported cases of cell surface CSPGs functioning in endocytosis are limited to low-density lipoprotein^18^, penetratin-directed CPPs^19^, human herpes simplex virus^20^, and *Clostridium difficile* toxin B^21^.

Here, we aimed to elucidate the function of both classes of cell surface HSPGs and CSPGs as true endocytic receptors for a basic recombinant anti-nucleic acid antibody (3D8 single-chain variable fragment (scFv); pI value, 9.15) that is internalized by a variety of living cells. Previous studies suggest involvement of HSPGs in 3D8 scFv endocytosis, although evidence is scant^22,23^. Given that both HS and CS are highly negatively charged GAGs (due to acidic sugar residues and/or modification by sulfate groups), we were motivated to study whether both HSPGs and CSPGs function as true endocytic receptors for 3D8 scFv (used as a basic model macromolecule). To address this, we investigated the interaction between 3D8 scFv and HSPGs and CSPGs, the effect of the treatment with soluble competitor HS/CS chains and HS/CS-removing enzymes on the binding of 3D8 scFv to cell surfaces and internalization of 3D8 scFv, the effect of removing sulfation from HS chains on the binding and internalization of 3D8 scFv, and the preferential use of GAG chains for the binding and internalization of 3D8 scFv.Moreover, we investigated whether both transmembrane and GPI-anchored HSPGs and CSPGs can function as an endocytic receptor on HeLa cells and HEK293 by transfecting cells with plasmids encoding individual HSPG and CSPG core proteins (SDC2, GPC3, CD44, and BCAN).

## Results

### 3D8 scFv binds to anionic HS and CS chains of HSPGs and CSPGs at the cell surface

**Figure 1:**
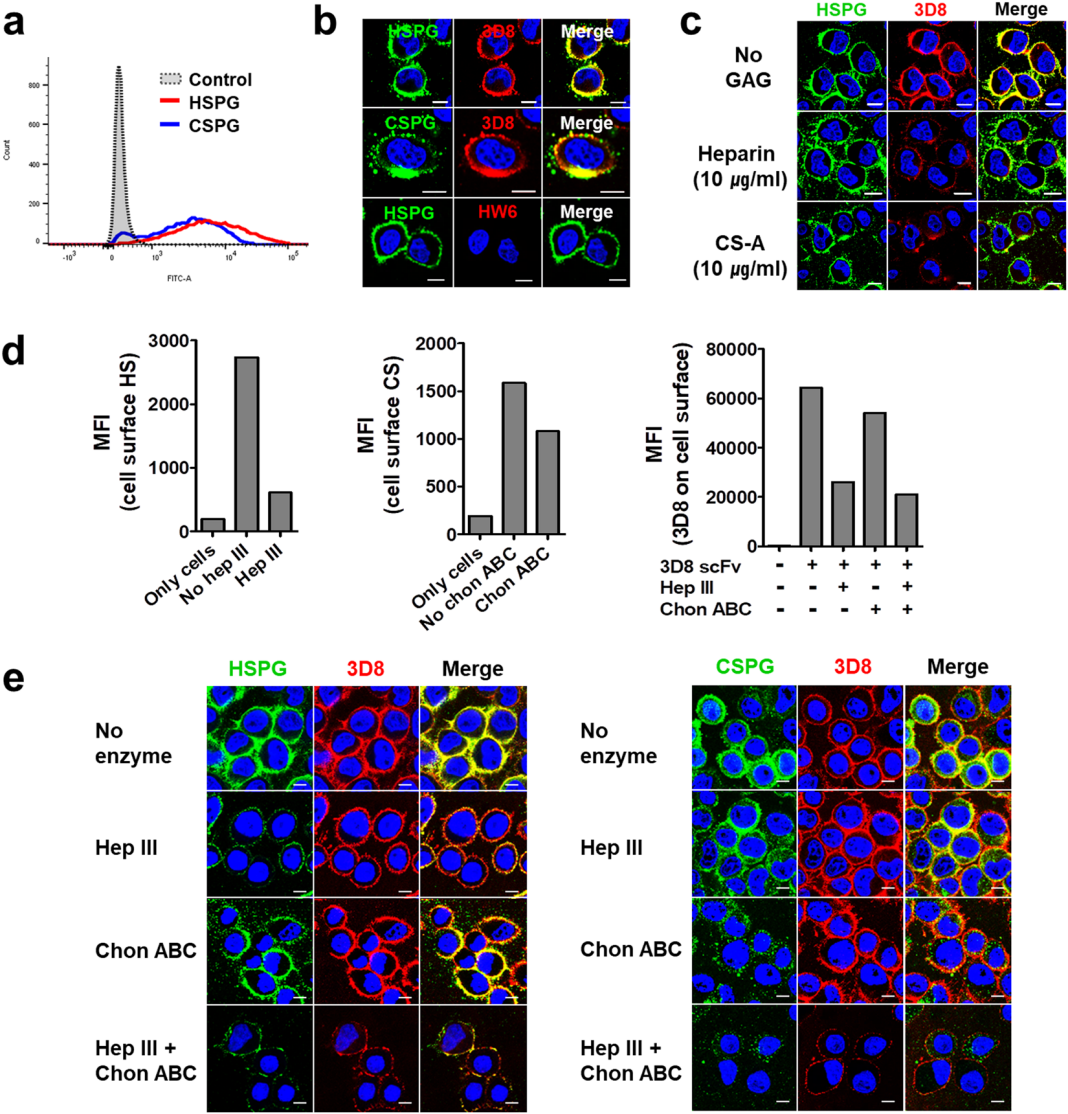
3D8 scFv binds to cell surface HSPGs and CSPGs. (**a**) Flow cytometry analysis of cell surface expression of endogenous HSPGs and CSPGs in HeLa cells. (**b**) Confocal microscopy to detect 3D8 scFv binding to cell surface HSPGs and CSPGs. HeLa cells were incubated for 1 h at 4 °C with 3D8 scFv (10 μM) and HW6 scFv (10 μM) (negative control). Thereafter, cells were incubated with a primary antibody mixture containing a rabbit anti-3D8 scFv antibody and a mouse IgM anti-HS antibody, or a rabbit anti-3D8 scFv antibody and a mouse IgM anti-CS antibody. After washing, cells were incubated with a secondary antibody mixture comprising TRITC-conjugated anti-rabbit IgG and Alexa Fluor 488-conjugated anti-mouse IgM. (**c**) Confocal microscopy to detect 3D8 scFv binding to cell surface HSPGs in the presence of soluble HS and CS chains. HeLa cells were incubated for 1 h at 4 °C with 3D8 scFv (10 μM) in the absence (*upper panel*) or presence (*middle* or *lower panel*) of heparin (10 μg/ml) or CS-A (10 μg/ml), followed by the procedures described in (**b**). (**d,e**) Flow cytometry (**d**) and confocal microscopy (**e**) to detect the cell surface sugar chains (HS and CS) and cell surface binding of 3D8 scFv to HeLa cells pre-treated with heparinase III (10 mIU/ml) and chondroitinase ABC (100 mIU/ml). (**b,c,e**) Nuclei were stained with Hoechst 33342 (blue). *Bar*, 10 μm.

First, we confirmed expression of HSPGs and CSPGs on the surface of HeLa cells by flow cytometry (Fig. 1a). Then, we examined whether 3D8 scFv binds to HSPGs and CSPGs on the surface of HeLa cells by confocal microscopy. HeLa cells were treated with 3D8 scFv at 4 °C for 1 h to suppress endocytosis and then with antibodies against HS or CS chains. 3D8 scFv colocalized with HSPGs and CSPGs on the cell surface (Fig. 1b). A negative control, HW6 scFv, which recognizes TRAIL receptor 2, did not bind to HSPGs or CSPGs. Binding of 3D8 scFv to HSPGs on the cell surface was markedly reduced in the presence of heparin (a soluble analogue of HS)^2^ and CS-A (Fig. 1c, Table 1). Moreover, binding of 3D8 scFv to HS and CS on the cell surface was reduced in cells pre-treated with GAG-degrading enzymes (Fig. 1d,e). Treatment with 10 mIU/ml of heparinase III and 100 mIU/ml of chondroitinase ABC reduced HS and CS levels on HeLa cell membranes by 85% and 35%, respectively (left and middle panels of Fig. 1d). Treatment of HeLa cells with heparinase III (10 mIU/ml), chondroitinase ABC (100 mIU/ml), and both heparinase III (10 mIU/ml) and chondroitinase ABC (100 mIU/ml), reduced binding of 3D8 scFv by 60%, 15%, and 78%, respectively (right panel of Fig. 1d). Consistent with the flow cytometry results, confocal microscopy showed reduced binding of 3D8 scFv to cell surface after treatment with GAG-degrading enzymes (Fig. 1e). The data demonstrate that 3D8 scFv binds to both HS and CS chains on the cell surface.

**Table 1:** Soluble GAG chains used in this study

Heparins of different sulfation types	Abbreviations
de-*N*-sulfated/*N*-acetylated heparin	*NAc*
de-2-*O*-sulfated heparin	*de2O*
de-6-*O*-sulfated heparin	*de6O*
de-*N*-sulfated/*N*-acetylated and de-2-*O*-sulfated heparin	*de2ONAc*
de-*N*-sulfated/*N*-acetylated and de-6-*O*-sulfated heparin	*de6ONAc*
exclusively N-sulfated heparin	*NS only*
completely de-*O*/*N*-sulfated/*N*-acetylated heparin	*de2,6ONAc*
**Chondroitin sulfates**	
chondroitin sulfate A (GlcA-GalNAc-4-*O*-sulfate)	CS-A
chondroitin sulfate C (GlcA-GalNAc-6-*O*-sulfate)	CS-C
**Dermatan sulfate (IdoA-GalNAc-4-** ***O*** **-sulfate)**	DS

“de-” denotes removal.

“-sulfated” denotes –SO_4_.

### HSPGs and CSPGs colocalize intracellularly with internalized 3D8 scFv and caveolin-1 (a caveolar marker)

**Figure 2:**
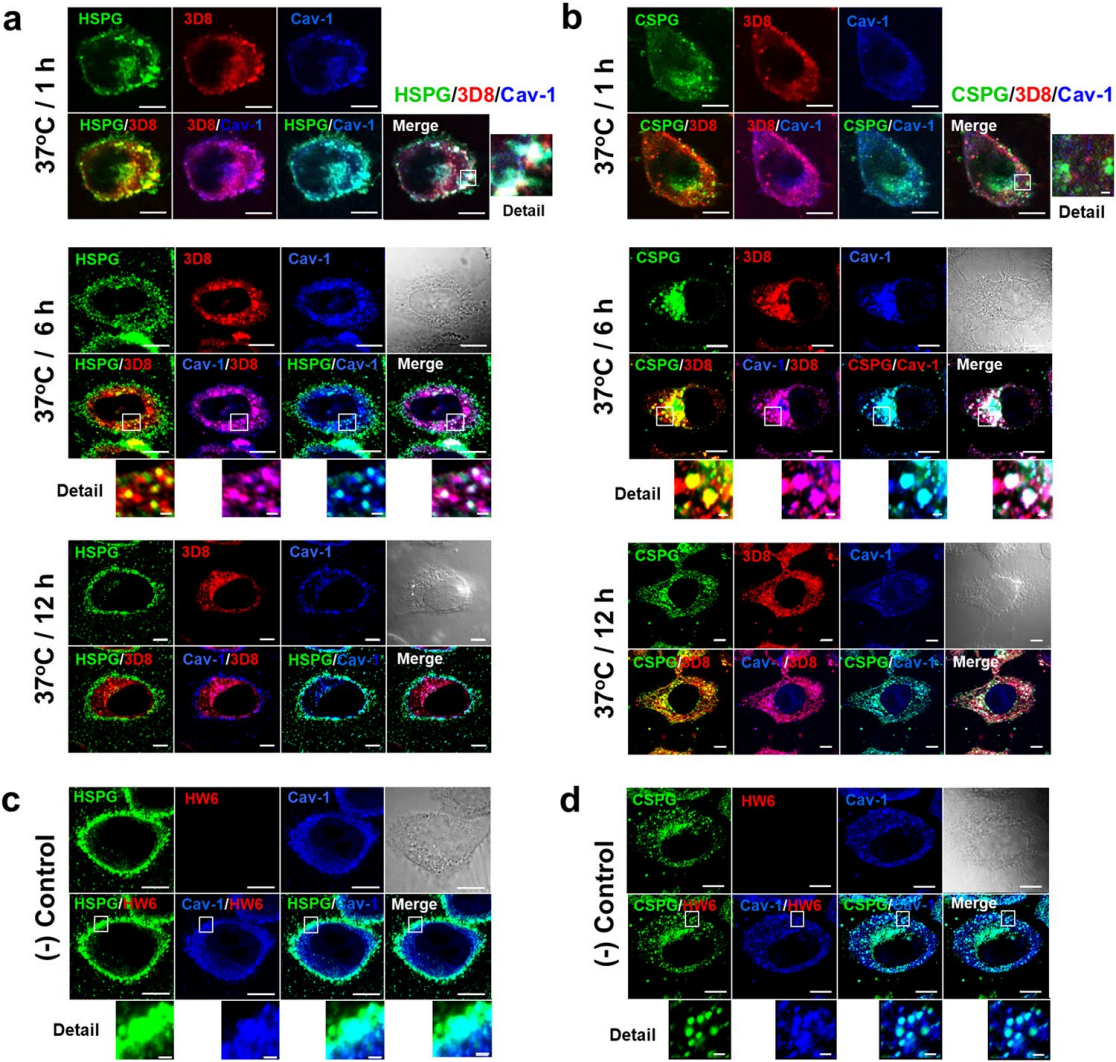
3D8 scFv colocalizes intracellularly with proteoglycans and caveolin-1 upon binding to cell surface proteoglycans (HSPGs and CSPGs). (**a,b**) Confocal microscopy to detect intracellular colocalization of 3D8 scFv, proteoglycans (HSPGs and CSPGs), and caveolin-1. HeLa cells were incubated with 3D8 scFv (10 μM) for 1 h, 6 h, or 12 h at 37 °C. After fixation and permeabilization, cells were incubated at 4 °C overnight with a primary antibody mixture comprising rabbit (IgG) anti-3D8 scFv, mouse (IgG) anti-caveolin-1, and mouse (IgM) anti-HS antibodies (**a**), or a mixture of rabbit anti-3D8 scFv, mouse anti-caveolin-1, and mouse (IgM) anti-CS antibodies (**b**). Thereafter, cells were incubated with a mixture of Alexa Fluor 647-conjugated goat anti-rabbit IgG, TRITC-conjugated goat anti-mouse IgG, and Alexa Fluor 488-conjugated goat anti-mouse IgM/μ chain-specific antibody. Enlarged images of the boxed areas in the upper panel are shown in the lower panel. (**c,d**) HeLa cells were incubated for 6 h at 37 °C with HW6, followed rabbit anti-His tag and Alexa Fluor 647-conjugated goat anti-rabbit IgG antibodies. *Bar*, 10 μm.

Our previous study showed that 3D8 scFv is internalized via the caveolae/lipid raft-mediated endocytic pathway and is finally released into the cytosol of HeLa cells without translocating to the endoplasmic reticulum, Golgi, or nucleus^23^. Accordingly, we speculated that if HSPGs and CSPGs act as true endocytic receptors for 3D8 scFv, they would colocalize intracellularly with 3D8 scFv and caveolin-1 (a caveolar structural protein). To investigate this, HeLa cells were incubated with 3D8 scFv for 1, 6, or 12 h at 37 °C and the intracellular localization of each molecule detected using specific antibodies and confocal microscopy. Fluorescence images revealed intracellular colocalization of 3D8 scFv and HSPGs (yellow), 3D8 scFv and caveolin-1 (magenta), HSPGs and caveolin-1 (cyan), and 3D8 scFv, HSPGs, and caveolin-1 (white) during endocytosis (Fig. 2a). Intracellular colocalization of 3D8 scFv and CSPGs (yellow), CSPGs and caveolin-1 (cyan), and 3D8 scFv, CSPGs, and caveolin-1 (white) during endocytosis was observed (Fig. 2b). At an early time point (1 h) after 3D8 scFv treatment, intensive colocalization of 3D8 scFv and HSPGs was observed both on the cell surface and inside the cells (Fig. 2a), whereas there was little colocalization of 3D8 scFv with CSPGs (Fig. 2b). 3D8 scFv, HSPGs, and caveolin-1 (Fig. 2a), as well as 3D8 scFv, CSPGs, and caveolin-1 (Fig. 2b), intensely colocalized after 6 h of 3D8 scFv treatment. This colocalization decreased after 12 h, indicating the spatial separation of these molecules. This tendency was also observed at lower magnification, and no cross-reactivity between mouse antibodies used for co-staining [anti-HS or anti-CS (mouse IgM antibodies) and the anti-caveolin (mouse IgG) antibody] was found (see Supplementary Fig. S1). This suggests that both HSPGs and CSPGs function as true endocytic receptors that direct 3D8 scFv to the caveosome-dependent endocytic pathway.

However, a closer inspection of Fig. 2a and b suggested that the colocalization of 3D8 with HSPGs and CSPGs was somewhat different. The vast majority of 3D8 no longer colocalized with cell surface and intracellular HSPGs at 6 and 12 h (very little red-green overlap); rather, it colocalized with caveolin-1 and so seemed to be internalized already. The majority of 3D8 at 6 and 12 h did not colocalize with cell surface CSPGs, but with intracellular CSPGs and caveloin-1, and so seemed to be still in the process of internalizing. Moreover, the majority of 3D8 scFv was spatially separate from HSPGs, but not from CSPGs, after 12 h. This suggests that HSPGs are the faster and primary endocytic receptor used for intracellular delivery of 3D8 scFv. In the absence of 3D8 scFv treatment, HSPGs and caveolin-1 mostly colocalized near/beneath the plasma membrane, whereas CSPGs and caveolin-1 mostly colocalized inside the cells (Fig. 2c,d). This probably represents their constitutive localization under normal conditions.

### Both transmembrane and GPI-anchored forms of HSPGs act as endocytic receptors for 3D8 scFv

**Figure 3:**
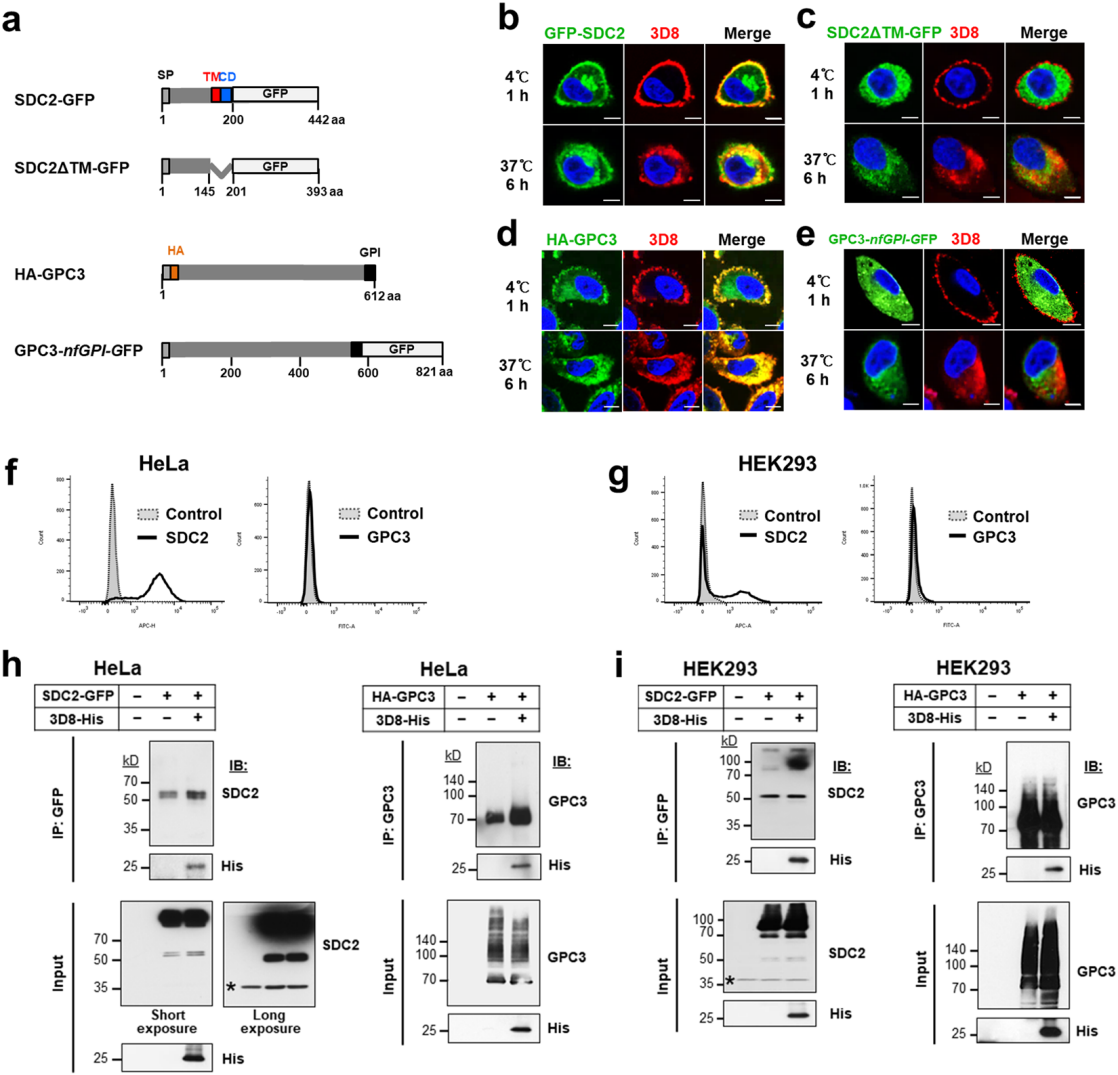
SDC2 and GPC3, representative HSPGs, act as endocytic receptors for 3D8 scFv on HeLa and HEK293 cells. (**a**) Schematic diagrams of SD2 and GPC3, which are expressed on the cell surface, and their recombinants, which are expressed intracellularly (controls). The parts of the core protein are shown without attached of GAGs. SP, signal peptide; Δ, deletion; TM, transmembrane domain; CD, cytoplasmic domain; GPI, GPI anchor; GFP, green fluorescent protein; HA, HA tag. (**b**–**e**) Confocal microscopy. HeLa cells were transfected with plasmids encoding SDC2-GFP (**b**) and HA-GPC3 (**d**) which are expressed on the cell surface, or with plasmids encoding SDC2ΔTM-GFP (**c**) and GPC3-*nf*GPI-GFP (*nf* indicates non-functional) (**e**), which are expressed intracellularly. At 18 h post-transfection, cells were incubated with 10 μM 3D8 scFv for 1 h at 4 °C or 6 h at 37 °C. Cells transfected with plasmids containing the GFP gene were incubated with a rabbit anti-3D8 scFv antibody, followed by a TRITC-conjugated goat anti-rabbit IgG antibody. Cells transfected with the plasmid encoding the HA-GPC3 gene were incubated with a rabbit anti-3D8 scFv antibody and a mouse anti-HA antibody, followed by TRITC-conjugated goat anti-rabbit IgG and Alexa Fluor 488-conjugated goat anti-mouse IgG, respectively. Nuclei were stained with Hoechst 33342 (blue). *Bar*, 10 μm. (**f**,**g**) Cell surface expression of endogenous SDC2 and GPC3 was examined by flow cytometry using core protein-specific antibodies. (**h**,**i**) Co-immunoprecipitation. HeLa (**h**) and HEK293 cells (**i**) were transfected with plasmids encoding SDC2-GFP and HA-GPC3 and treated for 6 h at 37 °C with 5 μM 3D8 scFv 24 h later. Cells were collected and lysed for co-immunoprecipitation assays. Co-immunoprecipitation was performed using an anti-GFP antibody. Samples were analyzed by immunoblotting with antibodies specific for SDC2 and the His tag (left panels of h, i). Samples from the co-immunoprecipitation performed using the anti-GPC3 antibody were analyzed by immunoblotting with antibodies specific for GPC3 and the His tag (right panels of h, i). Cells not treated with the 3D8 scFv protein were used as a negative control. Proteins in the extract (Input; 10%) and pulled-down fractions (IP) were analyzed by immunoblotting. Asterisks denote endogenous SDC2.

We questioned whether both transmembrane and GPI-anchored HSPGs act as endocytic receptors for 3D8 scFv. Two representative HSPGs, transmembrane SDC2 and GPI-anchored GPC3, were chosen. HeLa cells were transfected with the plasmids encoding GFP-SDC2 and HA-GPC3, which were expressed on the cell surface, and with the plasmids encoding SDC2ΔTM-GFP and GPC3-*nf*GPI-GFP, which were expressed intracellularly (Fig. 3a). At 18 h post-transfection, cells were treated with 3D8 scFv for 1 h at 4 °C, which is insufficient for internalization of 3D8 scFv, or for 6 h at 37 °C, which is favorable for internalization of 3D8 scFv. Thereafter, cells were observed by confocal microscopy. In cells incubated with 3D8 scFv for 1 h at 4 °C, 3D8 scFv colocalized (yellow) with SDC2 and GPC3 mostly on the cell surface (upper panels of Fig. 3b,d). However, 3D8 scFv did not colocalize with the intracellularly expressed forms of these molecules and they were observed as red and green fluorescence, respectively, separated by the cell membrane and localized inside or outside cells (upper panels of Fig. 3c,e). In cells incubated with 3D8 scFv for 6 h at 37 °C, internalized 3D8 scFv colocalized with cell membrane-associated SDC2 and GPC3 (lower panels of Fig. 3b,d). By contrast, internalized 3D8 scFv did not colocalize with the intracellularly expressed forms of these molecules and they were observed as red and green fluorescence, respectively, localized inside cells (lower panels of Fig. 3c,e).

Endogenous SDC2 was highly expressed on the cell surface of HeLa and HEK293 cells, whereas endogenous GPC-3 was not expressed at all (Fig. 3f,g). To determine whether HSPGs and 3D8 scFv interact physically, we transfected HeLa and HEK293 cells with plasmids encoding SDC2-GFP and HA-GPC3 and performed immunoprecipitation with anti-GFP and anti-GPC3 antibodies, respectively. Increased cell surface expression of HS chains and core proteins in transfectants was confirmed by flow cytometry (see Supplementary Fig. S2). SDC2-GFP and HA-GPC3 pulled down the 3D8 scFv from cell lysates (Fig. 3h,i), indicating an interaction between 3D8 scFv and SDC2 and GPC3. These data suggest that both transmembrane and GPI-anchored HSPGs are endocytic receptors for 3D8 scFv. The expression patterns of core proteins of HSPGs in HeLa and HEK293 cells were markedly different, resulting in detection of different band patterns for SDC2 and GPC3 in both the input and immunoprecipitated samples.

### Both transmembrane and GPI-anchored forms of CSPGs act as endocytic receptors for 3D8 scFv

**Figure 4:**
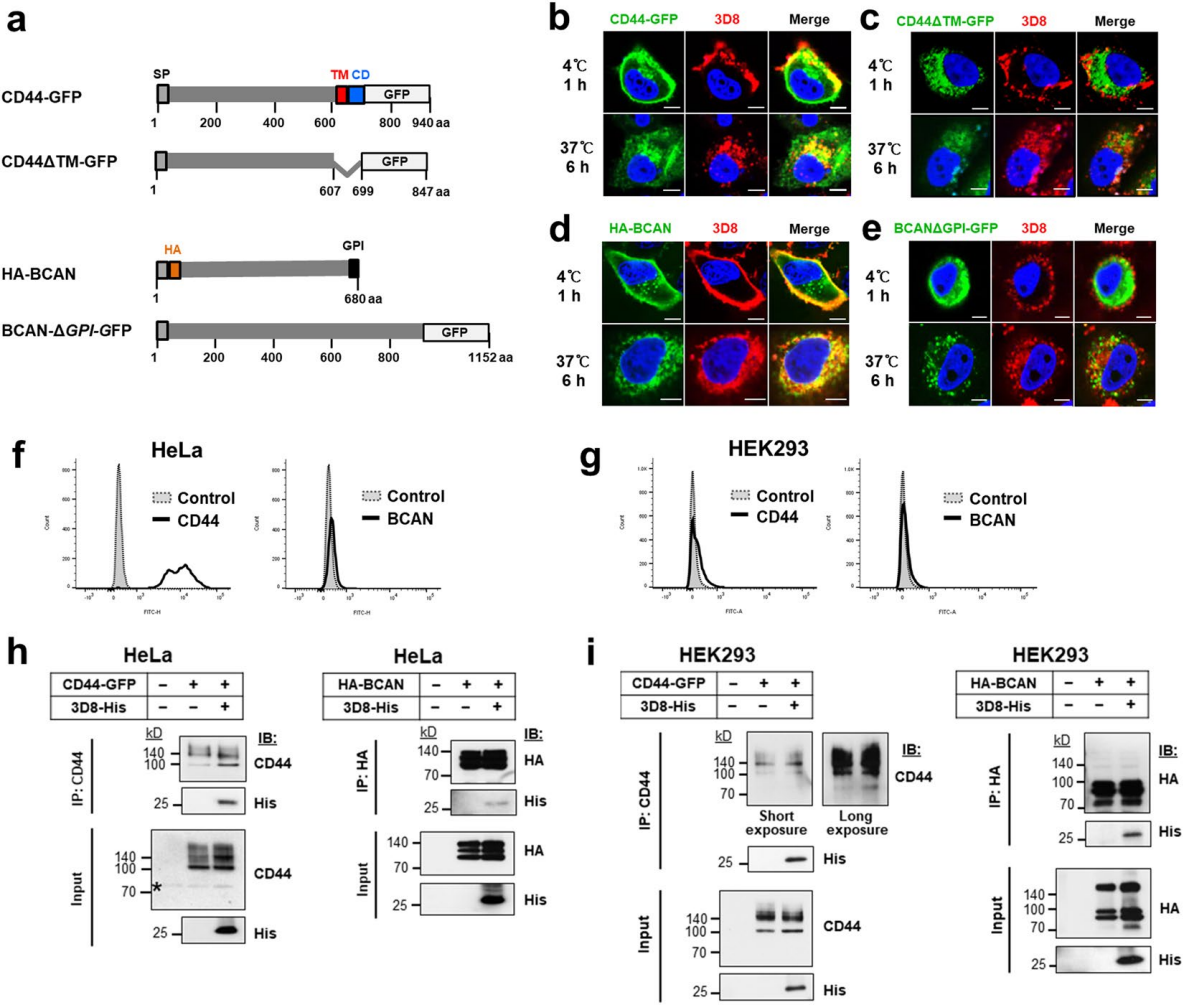
CD44 and BCAN, representative CSPGs, act as endocytic receptors for 3D8 scFv on HeLa and HEK293 cells. (**a**) Schematic diagrams of CD44 and BCAN, which are expressed on the cell surface, and their recombinants, which are expressed intracellularly (controls). SP, signal peptide; Δ, deletion; TM, transmembrane domain; CD, cytoplasmic domain; GPI, GPI anchor; GFP, green fluorescent protein; HA, HA tag. (**b**–**e**) Confocal microscopy. HeLa cells were transfected with plasmids encoding CD44-GFP (**b**) and HA-BCAN (**d**), which are expressed on the cell surface, or with plasmids encoding CD44ΔTM-GFP (**c**) and BCAN-ΔGPI-GFP (**e**), which are expressed intracellularly. At 18 h post-transfection, cells were incubated with 10 μM 3D8 scFv under the specified conditions. After fixation and permeabilization, cells transfected with plasmids containing the GFP gene were incubated with a rabbit anti-3D8 scFv antibody, followed by a TRITC-conjugated goat anti-rabbit IgG antibody. Cells transfected with the plasmid encoding the HA-BCAN gene were incubated with a rabbit anti-3D8 scFv antibody and a mouse anti-HA antibody, which were detected by TRITC-conjugated goat anti-rabbit IgG and Alexa Fluor 488-conjugated goat anti-mouse IgG, respectively. Nuclei were stained with Hoechst 33342 (blue). *Bar*, 10 μm. (**f**,**g**) Cell surface expression of endogenous CD44 and BCAN was examined by flow cytometry. (**h,i**) Co-immunoprecipitation. HeLa (**h**) and HEK293 cells (**i**) were transfected with plasmids encoding CD44-GFP and HA-BCAN and 24 h later treated with 5 μM 3D8 scFv for 6 h at 37 °C. Cells were collected and lysed for co-immunoprecipitation assays. Co-immunoprecipitation was performed using an anti-GFP antibody, Samples were analyzed by immunoblotting with antibodies specific for CD44 and the His tag (left panels of h, i). Samples from the co-immunoprecipitation performed with the anti-HA antibody, were analyzed by immunoblotting with antibodies specific for the HA tag and the His tag (right panels of h, i). Cells not treated with 3D8 scFv protein were used as a negative control. Proteins from the extract (Input; 10%) and pulled-down fractions (IP) were analyzed by immunoblotting. Asterisks denote endogenous CD44.

We examined whether both transmembrane and GPI-anchored CSPGs act as endocytic receptors for 3D8 scFv. Two representative CSPGs, transmembrane CD44 and GPI-anchored BCAN, were chosen. HeLa cells were transfected with plasmids encoding CD44-GFP and HA-BCAN, which were expressed on the cell surface, and with plasmids encoding CD44ΔTM-GFP and BCANΔGPI-GFP, which were expressed intracellularly (Fig. 4a). At 18 h post-transfection, cells were treated with 3D8 scFv for 1 h at 4 °C (which is insufficient for internalization of 3D8 scFv) or for 6 h at 37 °C. In cells incubated with 3D8 scFv for 1 h at 4 °C, 3D8 scFv colocalized (yellow) with CD44 and BCAN mostly on the cell surface (upper panels of Fig. 4b,d). However, 3D8 scFv did not colocalize with the intracellularly expressed forms of CD44 and BCAN, which were observed as red and green fluorescence, respectively, and were separated by the cell membrane and localized inside or outside cells (upper panels of Fig. 4c,e). In cells incubated with 3D8 scFv for 6 h at 37 °C, internalized 3D8 scFv colocalized with cell membrane-associated CD44 and BCAN (lower panels of Fig. 4b,d). By contrast, internalized 3D8 scFv did not colocalize with the intracellularly expressed forms of these molecules; these were observed as red and green fluorescence, respectively, localized inside cells (lower panels of Fig. 4c,e).

Endogenous CD44 was highly expressed on the HeLa cell surface but not on the HEK293 surface, whereas endogenous BCAN was not expressed by either cell type (Fig. 4f,g). To determine whether CSPGs and 3D8 scFv interact physically, we transfected HeLa and HEK293 cells with plasmids encoding CD44-GFP and HA-BCAN and performed immunoprecipitation with anti-CD44 and anti-HA antibodies, respectively. Increased expression of cell surface CS chains and core proteins by transfectants was confirmed by flow cytometry (see Supplementary Fig. S2). CD44-GFP and HA-BCAN pulled down 3D8 scFv from cell lysates (Fig. 4h,i), indicating an interaction between 3D8 scFv and CD44 and BCAN. These results suggest that both transmembrane and GPI-anchored CSPGs play a role as true endocytic receptors for 3D8 scFv. The expression patterns of core proteins of CSPGs in HeLa and HEK293 cells were markedly different, resulting in detection of different band patterns for CD44 and BCAN in both the input and immunoprecipitated samples.

### HS and CS chains are responsible for endocytosis of 3D8 scFv

**Figure 5:**
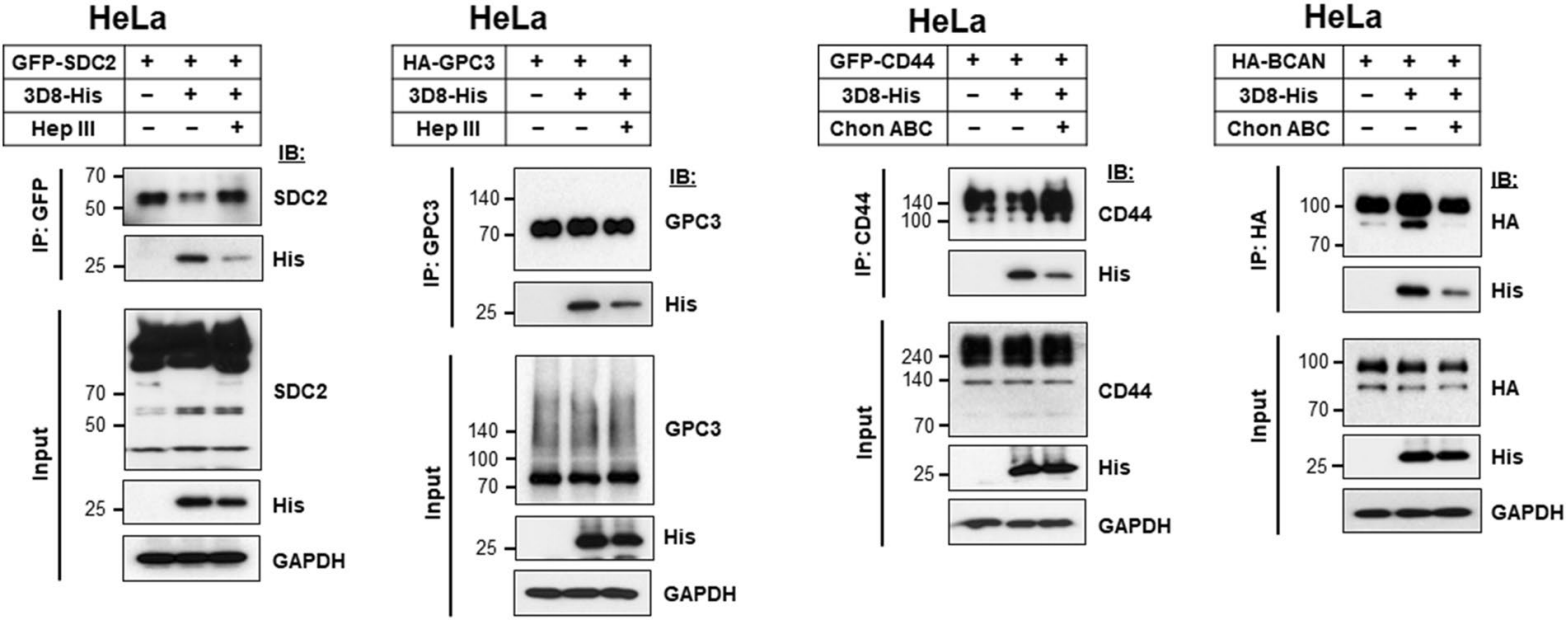
HS and CS chains are responsible for endocytosis of 3D8 scFv. At 24 h post-transfection with plasmids encoding SDC2-GFP, HA-GPC3, CD44-GFP, or HA-BCAN, HeLa cells were treated with 500 mIU/ml heparinase III or 500 mIU/ml chondroitinase ABC for 2 h at 37 °C. Then, cells were treated for 6 h at 37 °C with 3D8 scFv, followed by immunoprecipitation with anti-GFP, anti-GPC3, anti-CD44, or anti-HA antibodies. Proteins in the extract (Input; 10%) and pulled-down fractions (IP) were analyzed by immunoblotting.

The physical interaction between the GAG moieties (HS and CS chains) of HSPGs and CSPGs and 3D8 scFv was confirmed using GAG-degrading enzymes. HeLa cells transfected with plasmids encoding SDC2-GFP and HA-GPC3 were treated with heparinase III, and HeLa cells transfected with plasmids encoding CD44-GFP and HA-BCAN were treated with treated chondroitinase ABC, for 2 h at 37 °C. Next, cells were exposed to 3D8 scFv for 6 h at 37 °C and then immunoprecipitated with anti-GFP, anti-GPC3, anti-CD44, and anti-HA antibodies. Significantly less 3D8 scFv was pulled down from cells treated with GAG-degrading enzymes than from untreated controls, indicating an interaction between 3D8 scFv and HS and CS chains (Fig. 5).

### 3D8 scFv has a preference for HS chains over CS chains in binding and endocytosis

**Figure 6:**
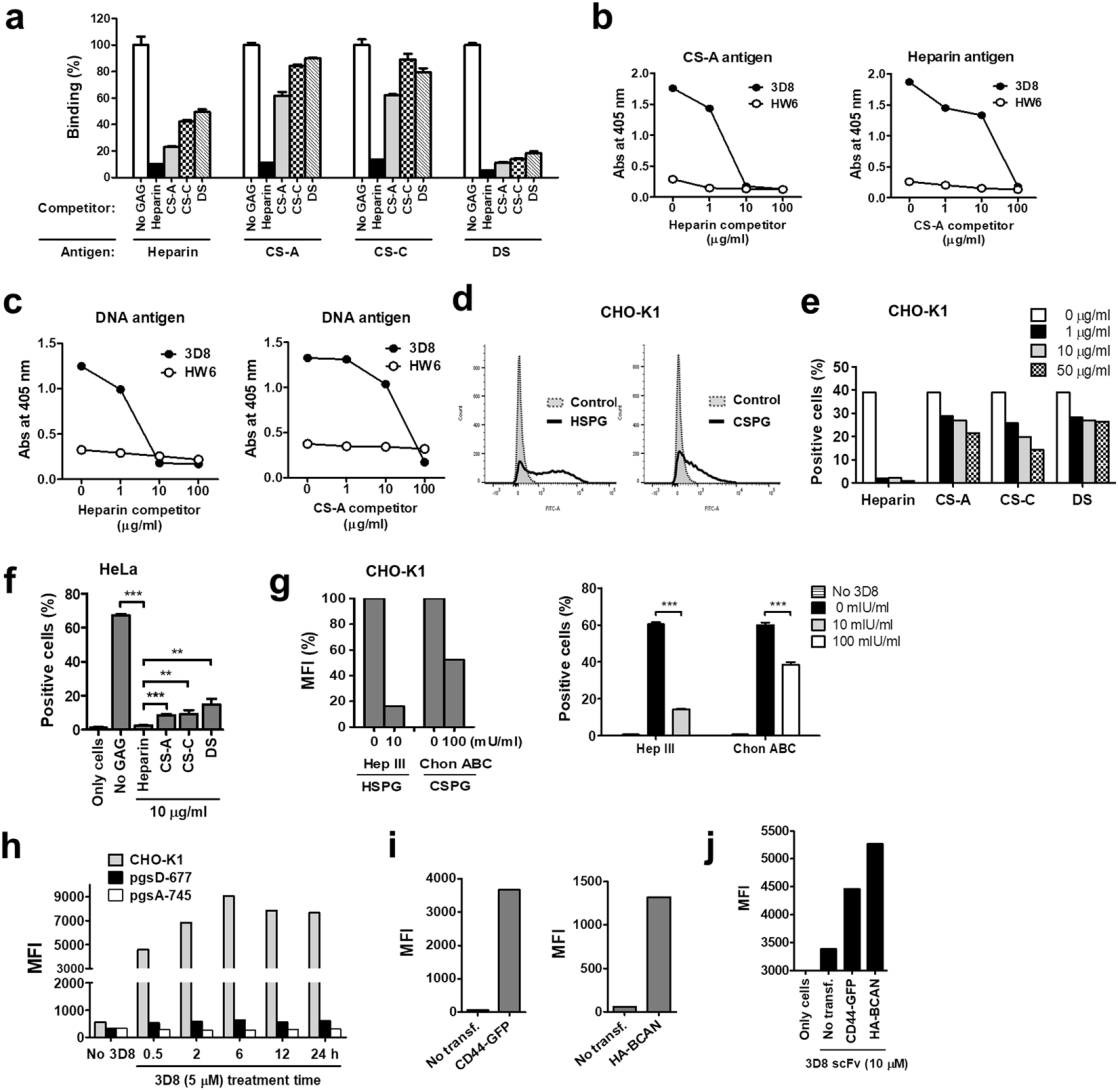
3D8 scFv prefers HS chains over CS and DS chains for binding and cellular internalization. (**a**) Competitive ELISA to determine the binding preference of 3D8 scFv for GAG chains. ELISA plates coated with 10 μg/ml soluble HS, CS-A, CS-C, or DS were incubated for 1 h at RT with 1:1 (v/v) mixtures of 3D8 scFv-pA (20 μg/ml) and each competitor (20 μg/ml), followed by detection of bound 3D8 scFv. (**b**,**c**) Competitive ELISA to compare the binding preference of 3D8 scFv between HS and CS. ELISA plates coated with 10 μg/ml of soluble CS-A, heparin (**b**), or 2 μg/ml of pcDNA3.1 plasmid DNA (**c**), were incubated with 1:1 (v/v) mixtures of 3D8 scFv-pA (20 μg/ml) and various concentrations (1–100 μg/ml) of competitors (heparin and CS-A, respectively). Binding of 3D8 scFv-pA to each antigen in the presence of competitors was determined using rabbit IgG, followed by AP-conjugated anti-rabbit IgG. Data represent the mean ± S.E. of triplicate wells and are representative of three independent experiments (**a–c**). (**d**) Flow cytometry to examine expression levels of endogenous HSPGs and CSPGs on CHO-K1 cell surface using antibodies specific for HSGPs and CSPGs. (**e**,**f**) Flow cytometry to examine internalization of 3D8 scFv in the presence of soluble GAGs. CHO-K1 (**e**) and HeLa cells (**f**) were incubated for 6 h at 37 °C with mixtures of 3D8 scFv (10 μM) and each soluble GAG (at the specified concentrations) (**g**) Flow cytometry to examine cell surface expression of HSPGs and CSPGs on CHO-K1 cells after enzyme treatment for 1 h at 37 °C (left panel) and the amount of internalized 3D8 scFv after enzyme treatment for 6 h at 37 °C (right panel). (**h**–**j**) Flow cytometry to detect internalization of 3D8 scFv by CHO cell lines treated with 3D8 scFv (5 μM) for 0.5–24 h at 37 °C (H), expression of CD44-GFP and HA-BCAN in pgsD-677 cells transfected with plasmids (I), and internalized 3D8 scFv in transfected pgsD-677 cells (**j**). A minimum of 10,000 cells per sample were analyzed. MFI, Mean Fluorescence Intensity. Data are expressed as the mean ± standard error of three independent experiments. All *p* values were calculated using a two-tailed Student’s t test. Statistical significance is indicated on the graphs (**p < 0.01; ***p < 0.001).

We investigated the binding preference of 3D8 scFv among HS, CS, and DS chains using competitive enzyme-linked immunosorbent assays (ELISAs) in which GAGs such as heparin, CS-A, CS-C, and DS were used as antigens or competitors (Table 1). DS is the repeating disaccharide unit of IdoA-GalNAc-4S. Heparin is a high-sulfated structural analogue of HS, in which IdoA (2 S)-GlcNS (6 S) is most abundant^2^. Binding of 3D8 scFv to four GAG antigens was inhibited in the presence of four GAG competitors to varying extents, with the heparin competitor showing the strongest inhibition (Fig. 6a). Binding of 3D8 scFv to GAG chains in order of strength was heparin > CS-A > CS-C > DS, indicating that 3D8 scFv has a binding preference for HS chains over other GAGs. The 4-O-sulfate group on GalNAc residues of CS had a strong effect on 3D8 scFv binding. In competitive ELISAs using varying concentrations of heparin and CS-A, binding of 3D8 scFv to CS-A was inhibited by 100% in the presence of 10 μg/ml heparin competitor. Binding of 3D8 scFv to the heparin antigen was inhibited at most by 30% in the presence of 10 μg/ml CS-A competitor and by 100% in the presence of 100 μg/ml CS-A (Fig. 6b). Binding of 3D8 scFv to DNA was more strongly inhibited by the heparin competitor than by the CS-A competitor (Fig. 6c). A negative control, HW6 scFv, did not bind to heparin or CS-A. This indicates that 3D8 scFv binds more strongly (~10-fold) to HS than to CS-A. Furthermore, we performed flow cytometry to compare the effect of soluble GAGs on endocytosis of 3D8 scFv. HSPGs and CSPGs were expressed on Chinese hamster ovary (CHO)-K1 cells (Fig. 6d). 3D8 scFv endocytosis by CHO-K1 cells was most strongly inhibited by heparin, a finding consistent with the binding preference (Fig. 6e). The strongest inhibitory effect of the heparin competitor on 3D8 scFv endocytosis was observed in HeLa cells (Fig. 6f).

We also investigated internalization of 3D8 scFv by CHO-K1 cells treated with GAG-degrading enzymes. Removal of HS and CS chains from CHO-K1 cell membranes was first assessed by treatment with increasing amounts of heparinase III or chondroitinase ABC for 1 h. The amounts of HS and CS chains were reduced by 84% and 48% respectively, after treatment with heparinase III (10 mIU/ml) and chondroitinase ABC (100 mIU/ml) (left panel of Fig. 6g). Internalization of 3D8 scFv by CHO-K1 cells treated with heparinase III (10 mIU/ml) was reduced by 76%, whereas that by cells treated with of chondroitinase ABC (100 mIU/ml) was reduced by 36% (right panel of Fig. 6g). Internalization of 3D8 scFv fell at 0.5, 2, 6, 12, 24 h by 88–93% in pgsD-677 cells, which are HS-deficient but still synthesize CS and DS chains, and by 94–97% in *pan*-GAG-deficient pgsA-745 cells (Fig. 6h). To confirm that CSPGs act as an endocytic receptor for 3D8 scFv despite a low preference for 3D8 scFv, we transfected pgsD-677 cells with CD44 and BCAN (Fig. 6i) and then exposed them to 3D8 scFv. Internalization of 3D8 scFv by pgsD-677 cells was higher in transfected pgsD-677 cells than in untransfected cells (Fig. 6j). Taken together, these data suggest that 3D8 scFv is internalized via HSPGs and CSPGs, but shows a preference for HS chains over CS chains for endocytosis into HeLa and CHO cells. The results are consistent with those shown in Fig. 2 suggesting that HSPGs are the primary endocytic receptors for 3D8 scFv.

### O-sulfate groups, rather than N-sulfate groups, on HS are important for heparin binding and internalization of 3D8 scFv

**Figure 7:**
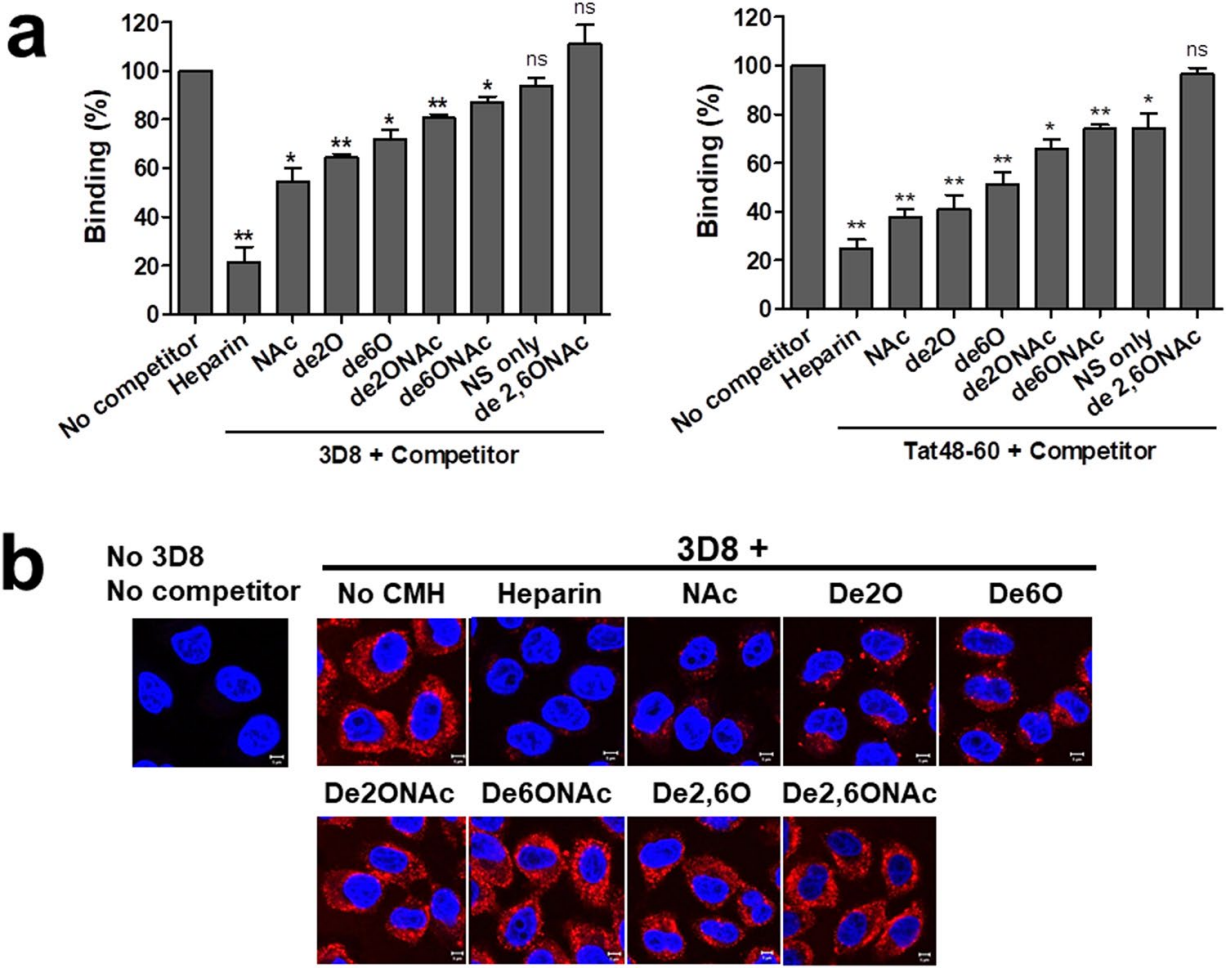
Sulfation of HS chains is important for HS binding and endocytosis of 3D8 scFv. (**a**) Binding of 3D8 scFv and the Tat peptide to heparin and CMHs and (**b**) internalization of 3D8 scFv were analyzed in the presence of heparin and CMHs. (**a**) Competitive ELISA for heparin binding. A microtiter plate was coated with heparin (10 µg/ml) and binding of 3D8 scFv-pA or the Tat peptide to heparin in the presence of CMHs (20 μg/ml) was determined using rabbit IgG, followed by detection with AP-conjugated anti-rabbit IgG (**a**) and AP-conjugated streptavidin, respectively. Data represent the mean ± S.E. of triplicate wells and are representative of three independent experiments. (**b**) Confocal microscopy analysis of 3D8 scFv internalization. CHO-K1 cells were incubated) for 6 h at 37 °C with a 1:1 mixture of 3D8 scFv-pA (20 μg/ml) and CMHs (20 μg/ml). After fixation and permeabilization, cells were stained with rabbit IgG, followed by TRITC-conjugated goat anti-rabbit IgG. Nuclei were stained with Hoechst 33342 (blue). *Bar*, 5 μm. Data are expressed as the mean ± standard error of three independent experiments. All *p* values were calculated using a two-tailed Student’s t test. Statistical significance is indicated on the graphs (ns, *p* > 0.05; *p < 0.05; **p < 0.01).

To investigate the effect of sulfation depletion on 3D8 scFv internalization, we performed competitive ELISAs using the chemically-modified heparins (CMHs) described in the Table 1. In parallel with 3D8 scFv, we also performed competitive ELISAs using the cell-penetrating peptide Tat as a positive control; this is because the Tat peptide requires for 2-O-, 6-O-, and N-sulfate groups for interaction with heparin^24^. Binding of 3D8 to heparin was not affected by CMHs lacking all O- and N-sulfate groups (de2,6ONAc) or all O-sulfates (NS only), indicating the importance of O-sulfation for the interaction between 3D8 scFv and HS. Removal of N-sulfate/2O-sulfate and N-sulfate/6O-sulfate (de2ONAc and de6ONAc, respectively) from heparin reduced competition by 10%, and individual removal of 2-O- or 6-O-sulfates (de2O and de6O, respectively) from heparin reduced competition by 20%, indicating the importance of O-sulfation (both 2-O and 6-O sulfation) and N-sulfation for the interaction between 3D8 scFv and HS. Removal of all N-sulfates from heparin, but retention of O-sulfation (NAc), reduced competition by 30%, in contrast to no reduction of CMHs lacking all O- and N-sulfate groups (de2,6ONAc) or all O-sulfates (NS only); this emphasizes that 3D8 scFv prefers to bind O-sulfate groups rather than N-sulfate groups on HS (left panel of Fig. 7a). Thus, sulfated HS chains play a critical role in the binding of positively charged 3D8 scFv. Binding of 3D8 scFv to heparin showed less dramatic, but similar levels of, inhibition by each CMH (left panel of Fig. 7a) when compared with binding of Tat peptide to heparin (right panel of Fig. 7a). As expected, a requirement for 2-O-, 6-O-, and N-sulfate groups for the Tat peptide-heparin interaction was observed. When we examined internalization of 3D8 scFv by CHO-K1 cells in the presence of soluble CMHs using confocal microscopy, we found that O-sulfations and N-sulfations were important for entry of 3D8 scFv into cells (Fig. 7b). Taken together, these results suggest that N- and O-sulfate groups on HS (which make it a strongly charged polyanion) are important for heparin binding by and subsequent endocytosis of 3D8 scFv. This result is similar to that reported by a previous study showing that SDCs and GPCs mediate uptake of an anti-HS antibody in a 2-O-sulfated HS-dependent but N-sulfated HS-independent manner^25^.

## Discussion

Here, we provide evidence that both cell surface HSPGs and CSPGs mediate intracellular delivery of the anti-nucleic acid (DNA and RNA) antibody 3D8 scFv, regardless of whether they are attached to the cell membrane via a transmembrane domain or a GPI anchor. This is the first evidence that both HSPGs and CSPGs are true endocytic receptors for a single macromolecule. Although the first direct evidence that HSPGs (both SDCs and GPCs) can function as true endocytic receptors, rather than co-receptors that only promote attachment and internalization of macromolecules, was provided by observation of the translocation of cell surface HSPGs to endocytic vesicles induced by an anti-HS scFv antibody^25^, no direct evidence that CSPGs are true endocytic receptors has been provided. Here, we observed that an internalizing 3D8 scFv antibody induced translocation of cell surface HSPGs and CSPGs to caveolin-1-containing endocytic vesicles (Fig. 2). Therefore, the study provides novel insight into the function of CSPGs as true endocytic receptors.

A subset of monoclonal anti-DNA antibodies are of biomedical interest because they are associated with autoimmune diseases in humans and mice, particularly systemic lupus erythematosus, and can be internalized by a variety of living cells^26–33^. Several receptors such as calreticulin, myosin 1, and equilibrative nucleoside salvage transporter (ENT) are suggested to be cell surface receptors for the internalization of some anti-DNA antibodies, although there is little direct evidence that they are endocytic receptors. For example, calreticulin^28^ and myosin 1^29^ were immuno-captured from cells treated with the monoclonal anti-DNA antibodies F14.6/H9.3 and H7, respectively. The anti-DNA monoclonal antibody 3E10 cannot penetrate ENT-deficient cells^34^. The 9D7 anti-dsDNA monoclonal antibody does not require a cell membrane receptor, even though it initially interacts with cell surface HSPGs for internalization^35^. Thus, it is worth noting that we first determined that both HSPGs and CSPGs function as endocytic receptors for an anti-DNA antibody (3D8 scFv). These previous findings and our present data suggest that different anti-DNA antibodies interact with different cell surface molecules for cellular internalization.

HSPG-mediated endocytosis is thought to be diverse and dependent on the cellular context and ligand type^3,7,36^. Usually, upon binding of ligands to sulfated domains within the HS chain, endocytosis of cell surface HSPGs via clathrin-coated pits or caveolae (and independent of clathrin, caveolin, and dynamin) occurs, although some ligands bind directly to HSPG core proteins. Proteins that bind to HS chains do not possess a specific fold or distinct amino acid sequence pattern, and the binding site for HS is often defined by positively charged residues in noncontiguous segments of the protein. This would be applicable to 3D8 scFv, in which several Lys and Arg residues are located in complementarity-determining regions. Our previous study showed that 3D8 scFv is endocytosed via a caveolin-dependent pathway, not by a clathrin-dependent or macropinocytotic pathway, escapes directly from the caveosome into the cytosol, and remains in the cytosol without further trafficking to endosomes, lysosomes, endoplasmic reticulum, Golgi, or nucleus in HeLa cells^23^. Here, we demonstrated that 3D8 scFv takes a caveosome-mediated pathway in HeLa cells, regardless of whether HSPGs or CSPGs act as endocytic receptors. Given that 3D8 scFv is endocytosed via various receptors, including at least two HSPGs (transmembrane SDC2 and GPI-linked GPC3) and two CSPGs (transmembrane CD44 and GPI-linked BCAN), it is surprising that only a caveolin-dependent pathway is used.

The characteristics of the ligand that binds to HSPGs/CSPGs may be the most important factor to determine the endocytosis route and intracellular trafficking itinerary, even if HSPGs and CSPGs simultaneously operate as endocytic receptors for cargo uptake. It remains to be determined how the endocytic pathway and the fate of internalized ligands depend on the ligand characteristics and how ligand interactions with transmembrane SDCs or GPI-linked GPCs can trigger endocytic signaling, although some signaling events through SDCs during endocytosis of ligands have been reported^7,8,37^.

Unlike 3D8 scFv, which uses both HSPGs and CSPGs as endocytic receptors, some ligands use specific HSPGs for endocytosis: SDC4 for liposome-medicated DNA transfer^38^ and CPPs such as penetratin, octaarginine, and TAT^8,39^, and SDC1 for baculovirus entry^10^ and lipoprotein uptake^40^. In these studies, however, the patterns of HS modification, the ligand-binding region on the core proteins of individual HSPGs, and the defined mechanisms are unclear. By contrast, NG2 (also known as CSPG4) has only been identified as the endocytic receptor for *Clostridium difficile* toxin B, and the binding region on the core protein of NG2 has been identified^21^. Positively charged 3D8 scFv binds to negatively charged HS and CS chains in HSPGs and CSPGs, respectively, for endocytosis, instead of binding to the core proteins. Based on the finding that 3D8 scFv has a preference for HS chains over CS chains for binding and endocytosis in HeLa and CHO-K1 cells, HSPGs seem to be a primary endocytic receptor for 3D8 scFv (Fig. 6). It is possible that expression levels of cell surface HSPGs and CSPGs, the amount of HS/CS chains, and the pattern/degree of sulfation of HS and CS chains might affect 3D8 scFv internalization into cells. The finding that 3D8 scFv uses both HSPGs and CSPGs as endocytic receptors also may be of interest because both CS and DS units may exist as hybrids on a core protein^1^, and SDC1 and SDC4 are hybrid-type proteoglycans that carry both HS and CS chains attached to the core protein, although they are classified as HSPGs^4,41^.

In conclusion, the collected data provide direct evidence that both HSPGs and CSPGs function as endocytic receptors for the uptake of a cationic anti-nucleic acid antibody. Our study may aid understanding of HSPGs and CSPGs as potential targets for macromolecular delivery and the entry route for macromolecules internalized through promiscuous receptors, and provides clues that may aid development of novel delivery strategies.

## Methods

### Cells

Wild-type CHO-K1 cells, HS-deficient CHO-K1 (ATCC^®^ CRL-2244) pgsD-677 cells, and *pan*-GAG-deficient CHO-K1 (ATCC^®^ CRL-2242) pgsA-745 cells were purchased from the American Type Culture Collection (ATCC). These cells were cultured in Ham’s F-12 medium (ATCC^®^ 30-2006) supplemented with 10% fetal bovine serum and antibiotics (100 U/ml penicillin and 100 μg/ml streptomycin). Human epithelial cervical carcinoma-derived HeLa cells and human embryonic kidney 293 cells (HEK293) were grown in Dulbecco’s modified Eagle’s medium containing the same supplements. Cells were incubated in a humidified incubator in 5% CO_2_ at 37 °C.

### scFv proteins and Tat peptide

The pIg20-scFv expression vector, in which the variable domain of the heavy chain (VH) and variable domain of the light chain (VL) are connected by a (Gly4/Ser1)_3_ linker after the Pho A leader signal sequence, was used to express scFv proteins with a (His)_5_ tag at the C-terminus. Otherwise, the pIg20-scFv-pA vector was used to express scFv-pA proteins with both a (His)_5_ tag and Staphylococcal Protein A (pA) at the C-terminus. ScFv proteins were expressed in *Escherichia coli* BL21(DE3) pLysE cells (Novagen)^42^ and purified from bacterial culture supernatants in a soluble form by Protein L-agarose affinity chromatography, according to the manufacturer’s guide (GE Healthcare Life Sciences, USA). The protein concentration was adjusted based on the molar extinction coefficient at 280 nm, which was calculated from the amino acid sequence. The HIV-Tat-derived peptide (amino acids 48–60 conjugated to biotin, [biotin-GRKKRRQRRRPPQ]) was purchased from Peptron (Daejeon, Korea).

### Plasmids encoding HSPGs and CSPGs

Human SDC2 and human GPC3, which are expressed on the cell surface via a transmembrane domain and GPI anchor, respectively, were chosen as representative membrane-associated HSPGs. Vectors for their expression on the cell surface and in the cytoplasm were purchased or constructed. The pCMV6-AC- SDC2-GFP vector used to express SDC2 on the cell surface as an N-terminal SDC2-GFP fusion protein was purchased from Origene (cat# RG203366). To express SDC2 in the cytoplasm, pEGFP-N3-SDC2ΔTM-EGFP was constructed by cloning the SDC2 gene lacking its transmembrane domain (SDC2ΔTM) into the pEGFP-N3 plasmid (Clontech) using the *EcoR*I and *Kpn*I restriction enzymes, resulting in expression of an N-terminal SDC2ΔTM-EGFP fusion protein. To express GPC3 on the cell surface as an N-terminal HA tag-GPC3 fusion protein, pEF-BOS-HA-GPC3 was purchased from Addgene (cat# 24670). To express GPC3 in the cytoplasm as an N-terminal GPC3-*nfGPI-G*FP fusion protein, pCMV6-AC-GPC3-turboGFP, in which the GPI anchor is non-functional, was purchased from Origene (cat# RG205911).

Human CD44 and human BCAN, which are expressed on the cell surface via a transmembrane domain and GPI anchor, respectively, were chosen as representative membrane-associated CSPGs. Vectors for their expression on the cell surface and in the cytoplasm were purchased or constructed. To express CD44 on the cell surface as an N-terminal CD44-turboGFP fusion protein, pCMV6-AC-CD44-turboGFP was purchased from Origene (cat# RG202455). To express CD44 in the cytoplasm, pCMV6-AC-CD44ΔTM-turboGFP was constructed by cloning the CD44 gene lacking its transmembrane domain into pCMV6-AC-CD44-turboGFP using the *EcoR*I and *Mlu*I restriction enzymes, resulting in expression of an N-terminal CD44ΔTM-turboGFP fusion protein. pCMV6-AC-BCANΔGPI-GFP was purchased from Origene (cat# RG205148) to express BCAN in the cytoplasm as an N-terminal BCANΔGPI-GFP fusion protein. To express BCAN on the cell surface, the HA tag and GPI anchor sequence plus a stop codon (NSACGSTALSILLLFFPLCLWVT-stop codon) were inserted into pCMV6-AC-BCANΔGPI-GFP at the N-terminus and C-terminus of the BCAN gene, respectively, resulting in expression of HA-BCAN.

### Confocal microscopy

The day before use, HeLa or CHO K-1 cells were seeded on glass coverslips in 24-well plates at a density of 5 × 10^4^ cells/well. Cells were then incubated with 3D8 scFv (10 μM) for 1 h at 4 °C, washed three times with cold phosphate-buffered saline (PBS, pH 7.2), and fixed by incubating with 4% paraformaldehyde (PFA) prepared in PBS for 10 min at room temperature (RT). Cells were incubated overnight at 4 °C with a primary antibody mixture comprising a rabbit anti-3D8 scFv antibody and a mouse anti-HS antibody (US Biological, cat# H1890, clone 10E4) or with a mixture comprising a rabbit anti-3D8 scFv antibody and a mouse anti-CS antibody (Abcam, cat# ab11570, clone CS56) diluted in buffer S (0.5% BSA and 2 mM EDTA prepared in PBS, pH 8.5). Negative control HW6 scFv cells were incubated with a rabbit anti-His tag antibody (Abcam, cat# ab9108), followed by Alexa Fluor 647-conjugated goat anti-rabbit IgG. After washing, cells were incubated with a secondary antibody mixture comprising TRITC-conjugated goat anti-rabbit IgG (Sigma, cat# T6778) and an Alexa Fluor 488-conjugated goat anti-mouse IgM/μ chain-specific antibody (Invitrogen, cat# A21042) diluted in surface buffer for 1–2 h at RT. Each incubation step was followed by three washes with cold PBS. Nuclei were stained with Hoechst 33342 (Thermo Fisher Scientific, cat# 62249) for the last 10 min of incubation at RT. After washing with cold PBS once, cells on coverslips were mounted in Vectashield anti-fade mounting medium (Vector Labs), observed with a Zeiss LSM 710 laser confocal microscope, and analyzed with Carl Zeiss LSM Image software.

To detect binding of 3D8 scFv to HSPGs or CSPGs on the cell surface in the presence of soluble GAGs, 3D8 scFv (10 μM) was pre-incubated for 30 min at 4 °C with soluble heparin (10 μg/ml) or CS-A (10 μg/ml). HeLa cells were then treated with each pre-incubated mixture for 1 h at 4 °C and fixed.

To detect binding of 3D8 scFv to HSPGs or CSPGs on the cell surface after treatment with GAG-degrading enzymes, HeLa cells were pre-treated with 10 mIU/ml heparinase III (Sigma, cat# H8891) or 100 mIU/ml chondroitinase ABC (Sigma, cat# C2905) for 1 h at 37 °C. Then, cells were treated with 3D8 scFv (10 μM) for 1 h at 4 °C, followed by fixation of the cell membrane.

To analyze the intracellular colocalization of 3D8 scFv, caveolin-1, and GAGs, HeLa cells were incubated with 3D8 scFv (10 μM) for 1, 6, or 12 h at 37 °C. Cells were washed three times for 10 min at RT with cold PBS, fixed with 4% PFA prepared in PBS, and then permeabilized by treatment with Perm-buffer (1% BSA, 0.1% saponin, and 0.1% sodium azide prepared in PBS) for 10 min at RT. After washing three times with cold PBS, cells were incubated overnight at 4 °C with a primary antibody mixture comprising rabbit anti-3D8 scFv, mouse anti-caveolin-1, and mouse anti-HS antibodies or rabbit anti-3D8 scFv, mouse anti-caveolin-1 (Santa Cruz, cat# sc-53564), and mouse anti-CS antibodies. After washing five times with cold PBS, cells were incubated for 1 h at RT with a secondary antibody mixture comprising Alexa Fluor 647-conjugated anti-rabbit IgG (Invitrogen, cat# A21244), TRITC-conjugated goat anti-mouse IgG (Santa Cruz, cat# sc-3796), and an Alexa Fluor 488-conjugated anti-mouse IgM/μ chain-specific antibody. All antibodies were diluted in Perm-buffer.

HeLa cells were transfected with HSPG (SDC2 and GPC3)- or CSPG (CD44 and BCAN)-encoding plasmids using Lipofectamine 2000 (Invitrogen), according to the manufacturer’s instructions. At 18 h post-transfection, cells were incubated for 1 h at 4 °C or for 6 h at 37 °C with 10 μM 3D8 scFv, fixed, and permeabilized. Cells transfected with plasmids encoding SDC2-GFP, SDC2ΔTM-GFP, GPC3-*nf*GPI-GFP, CD44-GFP, CD44ΔTM-GFP, or BCAN-ΔGPI-GFP were incubated with a rabbit anti-3D8 scFv antibody (produced in-house), followed by a TRITC-conjugated goat anti-rabbit IgG antibody. Cells transfected with plasmids expressing HA-GPC3 and HA-BCAN were incubated with a mixture comprising a rabbit anti-3D8 scFv antibody and a mouse anti-HA antibody, followed by a mixture of TRITC-conjugated goat anti-rabbit IgG and an Alexa Fluor 488-conjugated goat anti-mouse IgG antibody.

To examine the effect of CMHs on 3D8 scFv endocytosis, wild-type CHO-K1 cells were treated for 2 h or 30 min at 37 °C with a mixture of 3D8 scFv-pA (10 μM) and CMHs (20 μg/ml), fixed, permeabilized, and incubated with rabbit IgG (Sigma, cat#I5006), followed by TRITC-conjugated goat anti-rabbit IgG. CMHs were a generous gift from Dr. E. A. Yates (University of Liverpool) and were prepared as described previously^43^.

### Flow cytometry

To detect cell surface expression of HSPGs or CSPGs, cells were dissociated with non-enzymatic cell dissociation solution (Sigma, cat# C5914) and then treated with heparinase III (10 mIU/ml; Sigma, cat# H8891) or chondroitinase ABC (100 mIU/ml; Sigma, cat# C3367) for 1 h at 37 °C. After washing with serum-free transfection optimized medium (TOM) medium, cells were fixed for 10 min at RT with 4% PFA prepared in PBS. Then, cells were incubated with a primary mouse anti-HS antibody (US Biological, cat# H1890, clone 10E4) or a primary mouse anti-CS antibody (Abcam, cat# ab11570, clone CS56), followed by a secondary Alexa Fluor 488-conjugated goat anti-mouse IgM/μ chain-specific antibody (Invitrogen, cat# A21042). Finally, cells were examined by flow cytometry analysis. Each incubation (for 1 h at 4 °C) step was followed by three washes with cold PBS. To detect binding of 3D8 scFv to HSPGs or CSPGs on the cell surface after treatment with GAG-degrading enzymes, HeLa cells were pre-treated with heparinase III (10 mIU/ml) or chondroitinase ABC (100 mIU/ml) for 1 h at 37 °C. Then, cells were treated with 3D8 scFv (10 μM) for 1 h at 4 °C, followed by fixation of the cell membrane.

To detect cell surface expression of SDC2, GPC3, CD44, and BCAN, cells were fixed with 4% PFA without permeabilization, followed by incubation with mouse antibodies specific for GPC3 (Santa Cruz, cat# sc-65443), CD44 (Abcam, cat# ab6124), and BCAN (Abnova, cat# H00063827-B01P). Next, cells were incubated with secondary Alexa Fluor 488-conjugated goat anti-mouse IgG + IgM (Invitrogen, cat# A10680) or with a rabbit antibody against SDC2 (Bioss Inc., cat# bs-1904R), followed by Alexa Fluor 647-conjugated goat anti-rabbit IgG.

To examine the effect of GAG-degrading enzymes on 3D8 scFv internalization by CHO-K1 cells, cells were treated with heparinase III (10 mIU/ml) or chondroitinase ABC (100 mIU/ml) for 1 h at 37 °C. After washing with serum-free TOM medium, cells were incubated with 3D8 scFv for 6 h at 37 °C. The internalized 3D8 scFv was detected with a primary rabbit anti-3D8 scFv antibody, followed by TRITC-conjugated goat anti-rabbit IgG (Sigma, cat# T6778).

To detect internalization of 3D8 scFv-pA in the presence of soluble GAGs, HeLa and CHO-K1 cells were dissociated with non-enzymatic cell dissociation solution and resuspended in serum-free TOM medium. Thereafter, 1 × 10^6^ cells were incubated for 6 h at 37 °C with 3D8 scFv-pA (10 μM) in the presence of 1–50 μg/ml of each soluble GAG (heparin, CS-A, CS-C, and DS) dissolved in TOM medium, Cells were then subjected to the procedures described above. Heparin (cat# H3149), CS-A (cat# C9819), DS (cat# C3788), and CS-C (cat# 27043) were purchased from Sigma.

To detect internalization of 3D8 scFv-pA by wild-type CHO-K1, mutant pgsD-677 cells, and pgsA-745 cells, the cells were dissociated with non-enzymatic cell dissociation solution (Sigma, cat# C5914) and resuspended in serum-free TOM (Welgene). Thereafter, 1 × 10^6^ cells were incubated with 3D8 scFv-pA (5 μM) for 0.5–24 h at 37 °C. After washing, cells were treated for 5 min at 37 °C with 0.1% trypsin to remove surface-bound material and then fixed and permeabilized as described above. Cells were then incubated overnight at 4 °C with rabbit IgG. After washing three times with cold PBS, cells were incubated for 1 h at 4 °C with Alexa Fluor 647-conjugated anti-rabbit IgG. After washing, cells were resuspended in 4% PFA and analyzed by flow cytometry using a FACSCanto II cytometer (BD Biosciences).

To examine the effect of over-expressing CD44-GFP and HA-BCAN on 3D8 scFv internalization, pgsD-677 cells were transfected with plasmids encoding CD44-GFP and HA-BCAN. After 24 h, cells were incubated for 6 h at 37 °C with 3D8 scFv (10 μM), fixed, and permeabilized. The internalized 3D8 scFv was detected using rabbit anti-3D8 scFv and Alexa Fluor 647-conjugated anti-rabbit IgG. Over-expression of CD44-GFP in pgsD-677 cells was detected by measuring GFP fluorescence. Over-expression of HA-BCAN in pgsD-677 was detected with mouse anti-BCAN IgG (Abnova, cat# H00063827-B01P) and AlexaFluor 488-goat anti-mouse IgG (Invitrogen, cat# A10680).

### Competitive ELISA

To examine competition between different GAGs for binding to 3D8 scFv, 96-well ELISA plates were coated overnight at 4 °C with 10 μg/ml of each soluble GAG (HS, CS-A, CS-C, and DS) in PBS. The GAG-immobilized plates were incubated for 1 h at 37 °C with mixtures (1:1 v/v) comprising 20 μg/ml 3D8 scFv-pA and 20 μg/ml of each soluble GAG. After washing with TBST (Tris-buffered saline, 0.1% Tween 20), bound 3D8 scFv-pA was detected by a rabbit IgG antibody (Sigma, cat# I5006), followed by an alkaline phosphatase (AP)-conjugated goat anti-rabbit IgG antibody (Thermo Fisher Scientific, cat# 31341). Color was developed by adding ρ-NPP (ρ-nitrophenyl phosphate) substrate solution (1 mg/ml prepared in 0.1 M glycine, 1 mM ZnCl_2_, and 1 mM MgCl_2_, pH 10.3) to each well. Absorbance at 405 nm was read in a microplate reader (Molecular Devices).

To examine binding of 3D8 scFv to soluble GAG chains, ELISA plates coated with 10 μg/ml CS-A, heparin, or plasmid DNA antigen were incubated for 30 min at RT with mixtures (1:1 (v/v)) comprising 20 μg/ml 3D8 scFv-pA and 0–100 μg/ml heparin or CS-A competitor, followed by detection of bound 3D8 scFv as described above. To examine competition between 3D8 and soluble CMHs for binding to HS, ELISA plates coated with 10 μg/ml heparin were co-incubated for 1 h at 37 °C with mixtures (1:1 (v/v)) comprising 20 μg/ml 3D8 scFv-pA and 20 μg/ml of each CMH, followed by detection of bound 3D8 scFv as described above. To examine competition between the Tat_48-60_ peptide and CMHs for binding to HS, ELISA plates coated with 10 μg/ml heparin were co-incubated for 1 h at 37 °C with mixtures comprising biotinylated Tat_48-60_ (20 μg/ml) and each CMH (20 μg/ml). Bound Tat_48-60_ was detected by P-conjugated streptavidin (Invitrogen, cat# 43-4322).

### Immunoprecipitation (IP)

Cells were seeded in 6-well plates (3 × 10^5^ cells/well) the day before use. Cells were then transfected with plasmid (2.5 μg/well) using Lipofectamine 2000 (Invitrogen). At 24 h post-transfection, cell lysates were prepared using an IP kit (Thermo Cat# 26148), according to the manufacturer’s instructions. Briefly, after lysis with ice-cold IP Lysis buffer containing a protease inhibitor cocktail, 500 μg of cell lysate was subjected to IP with 2 μg of anti-GFP antibody (Abcam, cat# ab1218), anti-GPC3 antibody (Santa Cruz, cat# sc-65443), anti-CD44 (Abcam, cat# 6124), or anti-HA antibody (Millipore, cat# 05-904) coupled to a resin. If necessary, transfected HeLa cells were dissociated with non-enzymatic cell dissociation solution and treated with 500 mIU/ml heparinase III or 500 mIU/ml chondroitinase ABC for 2 h at 37 °C, followed by the treatment with 5 μM 3D8 scFv for 6 h at 37 °C. Then, cell lysates were prepared using the IP kit. Protein concentration in the cell lysates was measured using a BCA Protein assay kit (Thermo Cat# 23227). After washing the resin, the immunoprecipitated proteins were eluted and resolved on 4–20% gradient SDS-PAGE gels, followed by immunoblotting of proteoglycans and 3D8 scFv with antibodies specific for SDC2 (Invitrogen, cat# 36-6200), CD44 (Abcam, cat# 6124), GPC3, His tag (Abcam, cat# ab18184), or the HA tag.

## Electronic supplementary material

Supplementary Information
