# Supplementary material for: Heparan sulfate proteoglycans (HSPGs) and chondroitin sulfate proteoglycans (CSPGs) function as endocytic receptors for an internalizing anti-nucleic acid antibody
Source: Sci Rep. 2017 Oct 30;7:14373. doi: 10.1038/s41598-017-14793-z (PMC5662561; doi:10.1038/s41598-017-14793-z)
Supplement: Supplementary file 1 — Supplementary Information [file 41598_2017_14793_MOESM1_ESM.pdf]

Supporting information for

Heparan sulfate proteoglycans (HSPGs) and chondroitin sulfate proteoglycans (CSPGs) function as endocytic receptors for an internalizing anti-nucleic acid antibody

Hyunjoon Park[a,¶], Minjae Kim[a,¶], Hye-Jin Kim[a], Yeonjin Lee[a], Youngsil Seo[a], Chuong D. Pham[a], Joungmin Lee[a], Sung-June Byun[c], and Myung-Hee Kwon[a,b,*]

[a]Department of Biomedical Sciences, Graduate School, Ajou University, 206 World cup-ro, Yeongtong-gu, Suwon 443-749, South Korea.

[b]Department of Microbiology, Ajou University School of Medicine, 206 World cup-ro, Yeongtong-gu, Suwon 443-749, South Korea.

[c]Animal Biotechnology Division, National Institute of Animal Science, RDA, 1500, Kongjwipatjwi-ro, Iseo-myeon, Wanju-gun, Jeollabuk-do, 565-851, South Korea

*Correspondence to Myung-Hee Kwon: Department of Microbiology, Ajou University School of Medicine, Woncheon-dong 5, Suwon 443-749, Korea.
Tel.: +82-(31)-219-5074; FAX: +82-(31)-219-5079; E-mail: kwonmh@ajou.ac.kr.

¶These authors contributed equally to this work.

Supporting information contains:

Supplementary Figure 1 (Fig. S1) and legend

Supplementary Figure 2 (Fig. S2) and legend

Fig. S1

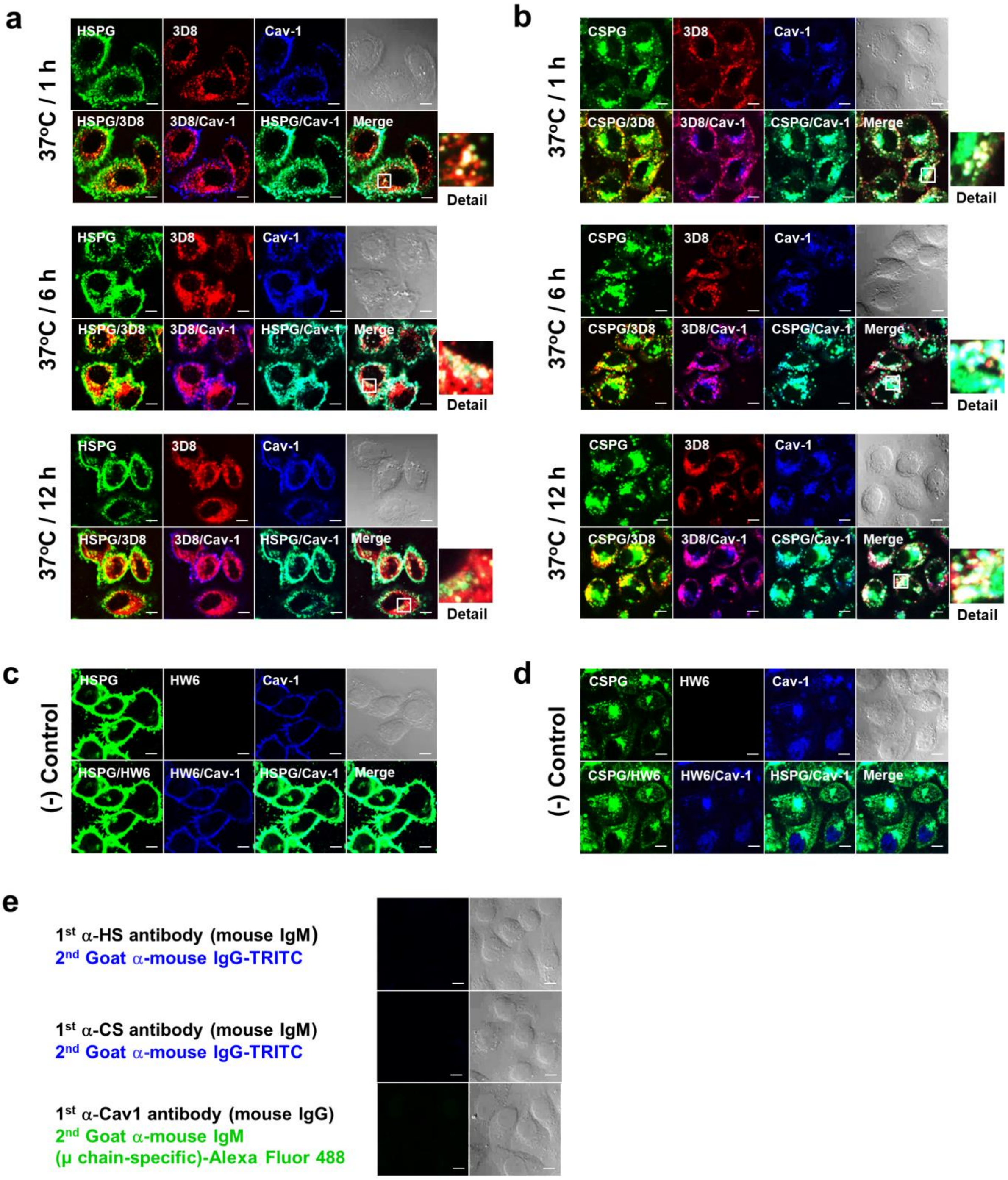

a
37°C / 1 h
HSPG
3D8
Cav-1
HSPG/3D8
3D8/Cav-1
HSPG/Cav-1
Merge
Detail
37°C / 6 h
37°C / 12 h
b
CSPG
CSPG/3D8
CSPG/Cav-1
c
(-) Control
HW6
HSPG/HW6
HW6/Cav-1
d
CSPG/HW6
e
1st α-HS antibody (mouse IgM)
2nd Goat α-mouse IgG-TRITC
1st α-CS antibody (mouse IgM)
2nd Goat α-mouse IgG-TRITC
1st α-Cav1 antibody (mouse IgG)
2nd Goat α-mouse IgM
(μ chain-specific)-Alexa Fluor 488

Supplementary Fig. S1. Intracellular colocalization of 3D8 scFv with proteoglycans (HSPGs and CSPGs) and caveolin-1. (a, b) Confocal microscopy was used to detect intracellular colocalization of 3D8 scFv, proteoglycans (HSPGs and CSPGs), and caveolin-1. HeLa cells were incubated for 1 h, 6 h, or 12 h at 37℃ with 3D8 scFv (10 μM). After fixation and permeabilization, cells were incubated overnight at 4℃ with a primary antibody mixture comprising rabbit (IgG) anti-3D8 scFv, mouse (IgG) anti-caveolin-1 (Santa Cruz, cat# sc-53564), and mouse (IgM) anti-HS (US biological, cat# H1890) antibodies (a), or with an antibody mixture comprising rabbit anti-3D8 scFv, mouse (IgG) anti-caveolin-1, and mouse (IgM) anti-CS (Abcam, cat# ab11570) antibodies (b). Thereafter, cells were incubated with a secondary antibody mixture comprising Alexa Fluor 647-conjugated goat anti-rabbit IgG (Invitrogen, cat# A21244), TRITC-conjugated goat anti-mouse IgG (Santa Cruz, cat# sc-3796), and Alexa Fluor 488-conjugated goat anti-mouse IgM/μ chain-specific antibodies (Invitrogen, cat# A21042). The boxed area is enlarged in the right panel. **(c, d)** HeLa cells were incubated for 6 h at 37℃ with HW6 (a non-internalizing scFv antibody used as a negative control), followed by incubation with rabbit anti-His tag and Alexa Fluor 647-conjugated goat anti-rabbit IgG. **(e)** Confocal microscopy of negative controls shows no cross-reactivity between mouse antibodies used for co-staining [anti-HS or anti-CS (mouse IgM antibodies) and the anti-caveolin (mouse IgG) antibody]. HeLa cells were fixed, permeabilized, and then incubated overnight at 4℃ with a mouse (IgM) anti-HS antibody (upper panel), a mouse (IgM) anti-CS antibody (middle panel), or a mouse (IgG) anti-caveolin antibody (lower panel). Thereafter, cells were incubated with TRITC-conjugated goat anti-mouse IgG (upper and middle panels) or an Alexa Fluor 488-conjugated goat anti-mouse IgM/μ chain-specific antibody (lower panel). *Bar,* 10 μm.

Fig. S2

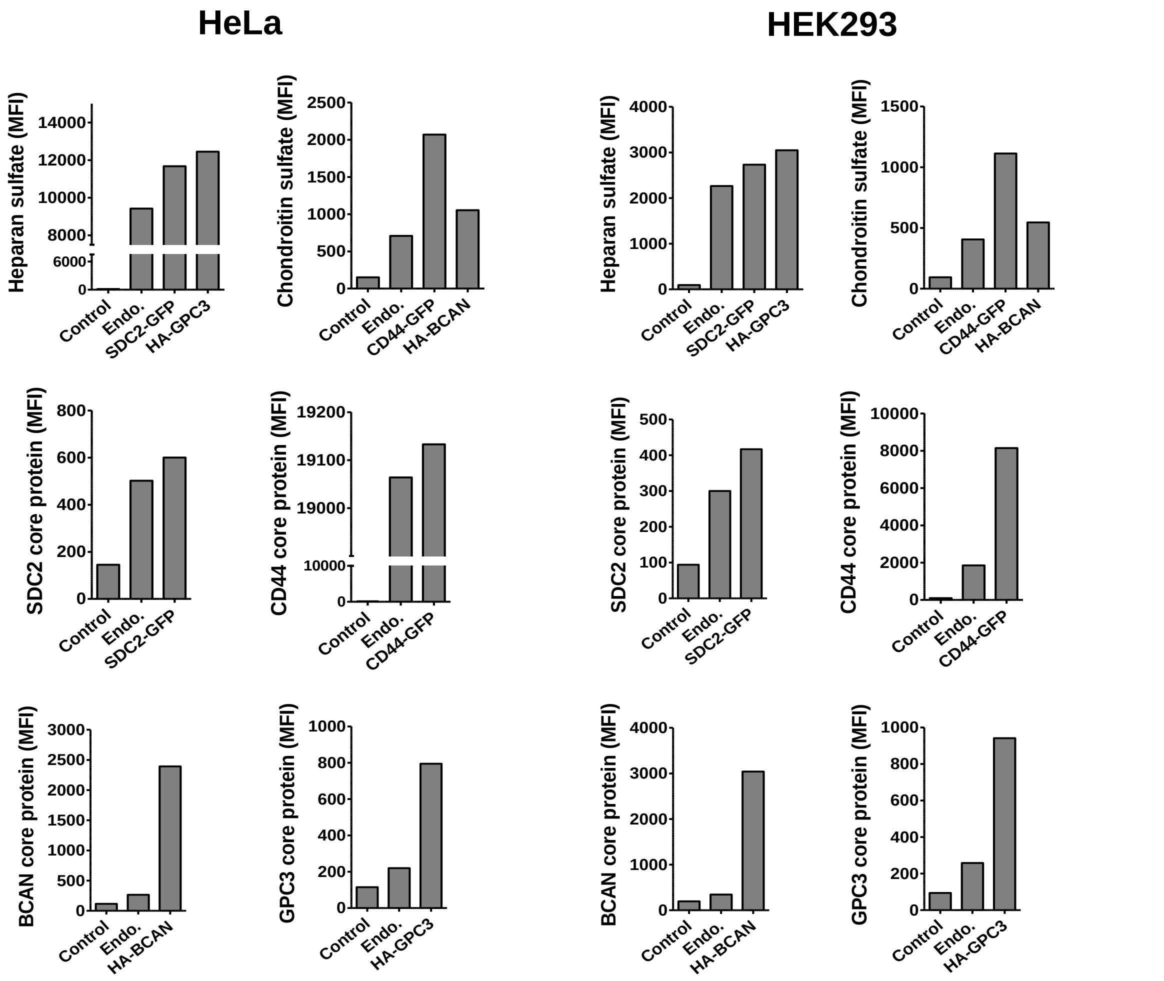

Supplementary Fig. S2. Flow cytometry to examine expression of GAGs and core proteins in cells transfected with HSPGs (SDC2 and GPC3) and CSPGs (CD44 and BCAN). HeLa and HEK293 cells were transfected with plasmids encoding SDC2-GFP, HA-GPC3, CD44-GFP, or HA-BCAN. After 24 h, the cells were dissociated with non-enzymatic cell dissociation solution (Sigma, cat# C5914), washed three times with cold phosphate-buffered saline (PBS), and fixed in 4% paraformaldehyde in PBS for 10 min at room temperature. In the experiment to detect increased expression of GAG by transfectants, cells were incubated overnight at 4°C with mouse anti-HS IgM (US Biological, cat# H1890) or mouse anti-CS IgM (Abcam, cat# ab11570) diluted in surface buffer (0.5% BSA and 2 mM EDTA prepared in PBS, pH 8.5). After washing, cells were incubated for 1 h at 4°C with an AlexaFluor 647-conjugated rat anti-mouse IgM/µ chain-specific antibody (BioLegend,

cat# 406526) diluted in surface buffer. In the experiment to detect increase expression of core protein by transfectants, cells transfected with plasmids expressing HA-GPC3 and HA-BCAN were incubated with mouse anti-GPC3 IgG (Santa Cruz, cat# sc-65443) or mouse anti-BCAN IgG (Abnova, cat# H00063827-B01P), followed by AlexaFluor 488-goat anti-mouse IgG (Invitrogen, cat# A10680). Cells transfected with plasmids expressing SDC2-GFP and CD44-GFP were incubated with mouse anti-SDC2 IgM (Santa Cruz, cat# sc-376229) or mouse anti-CD44 IgG (Abbexa, cat# abx19050), followed by an AlexaFluor 647-conjugated rat anti-mouse IgM/μ chain-specific antibody (BioLegend, cat# 406526). After washing, cells were resuspended in 4% paraformaldehyde and analyzed by flow cytometry using a FACSCanto II cytometer (BD Biosciences). SDC2, syndecan 2; GPC3, glypican 3; BCAN, brevican; MFI, mean fluorescence intensity.
